# Proactive Congestion Avoidance for Distributed Deep Learning

**DOI:** 10.3390/s21010174

**Published:** 2020-12-29

**Authors:** Minkoo Kang, Gyeongsik Yang, Yeonho Yoo, Chuck Yoo

**Affiliations:** Department of Computer Science and Engineering, Korea University, 145, Anam-ro, Seongbuk-gu, Seoul 02841, Korea; mkkang@os.korea.ac.kr (M.K.); yhyoo@os.korea.ac.kr (Y.Y.)

**Keywords:** distributed deep learning, P4, congestion avoidance, deep learning, network congestion, proactive congestion notification

## Abstract

This paper presents “Proactive Congestion Notification” (PCN), a congestion-avoidance technique for distributed deep learning (DDL). DDL is widely used to scale out and accelerate deep neural network training. In DDL, each worker trains a copy of the deep learning model with different training inputs and synchronizes the model gradients at the end of each iteration. However, it is well known that the network communication for synchronizing model parameters is the main bottleneck in DDL. Our key observation is that the DDL architecture makes each worker generate burst traffic every iteration, which causes network congestion and in turn degrades the throughput of DDL traffic. Based on this observation, the key idea behind PCN is to prevent potential congestion by proactively regulating the switch queue length before DDL burst traffic arrives at the switch, which prepares the switches for handling incoming DDL bursts. In our evaluation, PCN improves the throughput of DDL traffic by 72% on average.

## 1. Introduction

Distributed deep learning (DDL), which trains deep learning models using multiple workers, is gaining attention because it reduces the total training time of deep learning and is easy to scale. Due to these advantages, DDL is widely used by popular deep learning frameworks, including TensorFlow [1], MXNet [2], and PyTorch [3]. Although DDL implementations are different in each framework, the fundamental structure is as follows. Each worker trains a copy (replica) of a deep learning model using local training data and synchronizes the model parameters at the end of each training iteration. For synchronization, there are two common methods: (1) using a parameter server (PS) to collect and distribute parameters (PS architecture [4]) and (2) using all-to-all communication among workers (all-reduce architecture [5]). In both cases, workers send gradients and gather the updated model parameters.

Ideally, DDL should achieve a near-linear performance gain in proportion to the number of workers. However, because all workers have to synchronize their trained parameters at the end of each iteration, communications for parameter synchronization result in severe bottlenecks in DDL training, called “communication overhead” [6]. Thus, enhancing DDL communication is essential to accelerating the entire DDL training process because this communication time can occupy up to 92.8% of the total training time [7,8]. Therefore, finding solutions to communication overhead is an important research topic.

In an effort to overcome the communication overhead, we first analyze how each DDL worker generates traffic for synchronizations (DDL traffic) and measure the traffic characteristics using image classification models (e.g., ResNet [9], AlexNet [10], and VGG16 [11]) with TensorFlow. In PS architecture, the communication between the workers and the PS occurs right after the backpropagation of each worker is finished. Specifically, the worker sends the gradients as its training results to the PS, and the PS sends the updated model parameters to the workers. This characteristic of PS architecture makes network communication be concentrated in a short period. This communication can impact the performance on two sides in PS architecture: (1) memory input/output (IO)—for data communication between GPU memory and main memory (including PCIe bottlenecks); and (2) network—between worker nodes and a PS node. In terms of memory IO, GPU-vendor-driven solutions, such as hardware-based improvements, are being delivered (e.g., NVIDIA GPUDirect [12] and NCCL [13]). This paper focuses on network bottlenecks, which include the performance impact between the workers’ NIC and PS’s NIC, including the network switches between them.

To observe the network traffic characteristics, we conduct several measurements and observe that DDL traffic is periodically generated in bursts (Section 2.1). Specifically, the DDL system in our evaluation generates a large number of packets within very short intervals (e.g., milliseconds). This type of traffic burstiness is known to be the main cause of network congestion [14,15]. In other words, DDL burst traffic periodically makes the network congested, causing throughput degradation, high delay, and packet loss; thereby, the total training time increases. Because the DDL burst traffic results from the fundamental nature of the DDL itself, the DDL traffic inevitably causes network congestion. Recently, efforts [6,7,8,16,17,18] have been made to reduce the network communication bottleneck of DDL by changing the transmission order of deep neural network (DNN) layers to overlap and hide the communication overhead (Section 2.2). However, to the best of our knowledge, these efforts have not addressed the network congestion problems caused by the burstiness of DDL traffic.

In addition, existing congestion control schemes are not enough to address this problem. In conventional congestion control, the transport protocol (e.g., TCP) detects congestion after the packet is lost at the end host, so it takes longer to react to congestion. To reduce the reaction time to congestions, explicit congestion notification (ECN) is used to explicitly notify hosts of congestion based on the switch queue status without a packet drop [19]. Specifically, when the switch queue length is higher than a certain threshold, packets are marked with a congestion encountered (CE) bit in the TCP header fields (congestion marking). The host can slow down as soon as a congestion marked packet arrives at the host. This threshold is initially set by the network operators and usually has a range of up to 80, depending on the switch specification (Section 3.3). The end host receiving the congestion-marked packets reduces the TCP congestion window and decreases the sending rate so that the congestion can be cleared [20]. However, the ECN approach handles network congestion after burst traffic is generated; therefore, the existing congestion control approaches are inefficient for DDL communication because the congestion is identified after DDL generates burst traffic. This means that the ECN approach does not help reduce the congestion caused by the burstiness of DDL traffic.

As a solution, we propose proactive congestion notification (PCN), a novel congestion-avoidance technique. The key idea behind PCN is based on the following insight: the bustiness of DDL traffic can be known in advance. This is due to the fact that DDL consists of iterations for training and the time when DDL traffic is generated is known in each iteration. With the anticipation of DDL traffic, PCN attempts to regulate the traffic stacked on a switch in advance, so that network switches do not become congested when DDL traffic arrives. The PCN mechanism is to modify the congestion marking threshold, considering that burst traffic occurs periodically (details in Section 3.2).

To realize PCN, we use P4 [21] and programmable switches (Section 2.3) because traditional switches do not support the operations for PCN. For instance, PCN requires a way to pass the new threshold (PCN threshold) to the switches. Furthermore, a switch should be able to change its original threshold (initial threshold) to the new PCN threshold. However, traditional switches can only parse predefined types of network packets, and they cannot change the initial threshold. In contrast, using P4, a new type of packet header can be defined, and the desired bit of the header can be parsed and used in a programmable switch. Furthermore, new switch operations, such as match-action, can be implemented as needed for PCN (see details in Section 2.3). Recently, several studies tried to enhance DDL traffic by using an in-network switch for aggregating parameters or flow scheduling [22,23]. However, to the best of our knowledge, this is the first study that proactively handles the burstiness of DDL traffic via P4. We implement PCN functionalities in P4 and a traffic generator that simulates the common DDL traffic pattern observed with VGG16, AlexNet, and ResNet (Section 2.1). Then, we run and evaluate PCN on BMv2 [24], a P4 software switch. The evaluation results show an average 72% improvement in throughput (Section 4).

The remainder of this paper is organized as follows. Section 2 explains the background, related work, and motivation. Section 3 details the PCN design, and Section 4 presents the evaluation results. Section 5 discusses the limitations and future work of PCN. Finally, Section 6 concludes the paper.

## 2. Background and Motivation

### 2.1. Distributed Deep Learning Traffic

**Figure 1:**
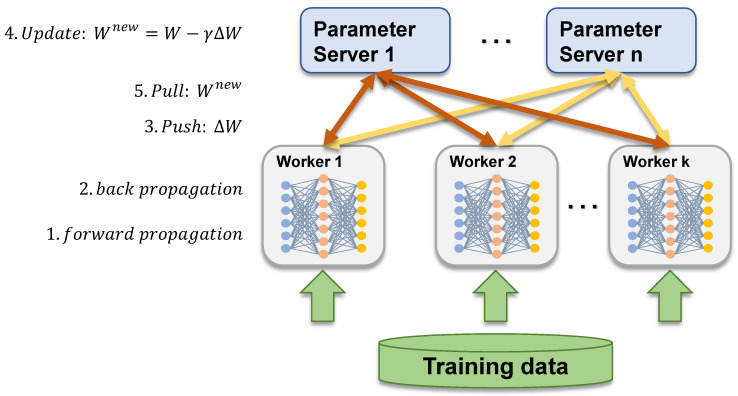
Parameter server architecture.

In DDL, network communication for parameter synchronization is known to be a major bottleneck [6,7,8]. Figure 1 depicts the training steps of the PS architecture, where the training operations (forward propagation and backpropagation) wait for communication operation (push and pull), and communication operation generates bursty DDL traffic. To verify the degree of DDL traffic burstiness, we measure the traffic generated by workers. We set our DDL environment with PS architecture because it is a popular approach for parameter synchronization. We use TensorFlow v1.6 without applying additional DDL optimizations (Section 2.2). The reason is that the optimizations deal with host-side communication that schedules the tensor transmission. Our paper focuses on the in-network congestion that is caused by the burst pattern of DL models. Note that the evaluation results shown here are similar to those of such studies, detailed in Section 2.2.1. We configure two workers and a single PS that communicate with each other using gRPC [25] over TCP. We train ResNet [9], AlexNet [10], and VGG16 [11] models using the ImageNet dataset [26] as the input, with a mini-batch size of 128 for all models. Stochastic gradient descent is used as an optimizer, and the framework configurations, i.e., mixed precision, use_fp16, are disabled by default. We use a server with two NVIDIA V100 GPUs that consist of 640 cores and supports 130 teraflops [27], a single V100 GPU is pinned to each worker, and NCCL [13] is not supported. We containerize the PS and workers using Docker [28], which makes experiments easily reproducible.

**Figure 2:**
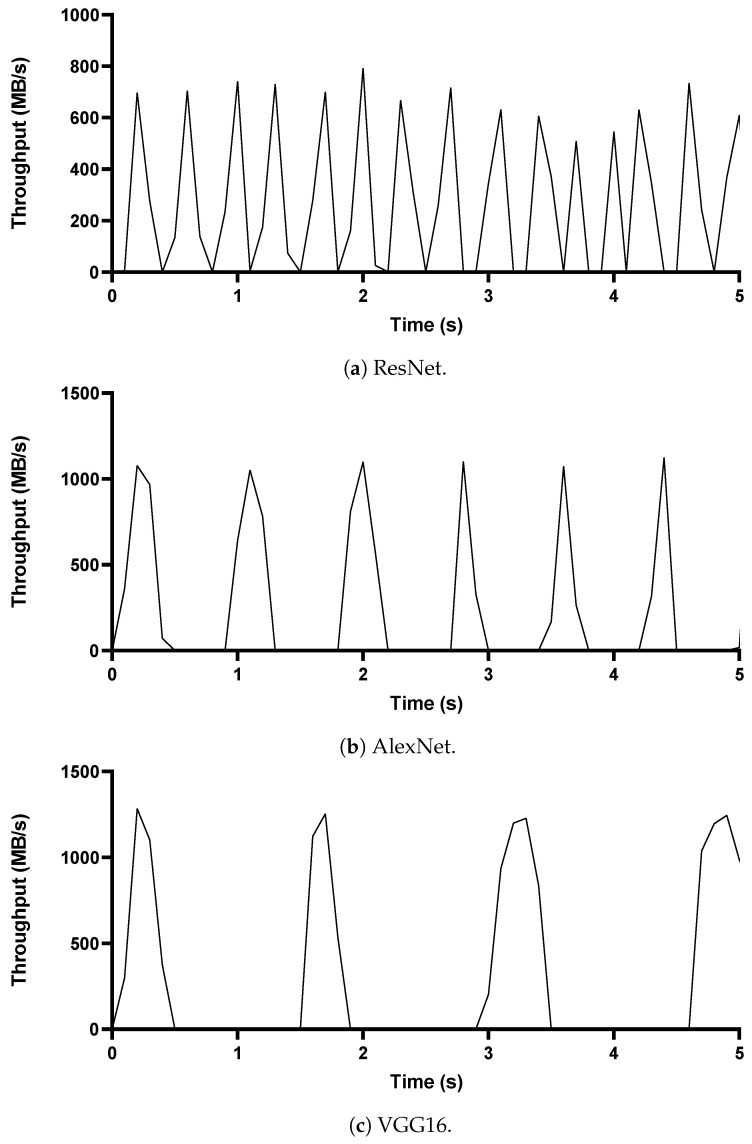
Communication patterns of deep learning models.

Figure 2 shows the experiment results for communication patterns; the y-axis shows the network throughput of the traffic generated by one worker over time (we only exhibit the throughput of a single worker because two workers in experiments show almost identical traffic patterns.). To measure the throughput, we use tcpdump [29] on the virtual interfaces of containers running workers and collect only DDL traffic from packet traces. These results (Figure 2a–c) of the three models demonstrate burst-idle traffic patterns in which a DDL worker stays idle in communication during training and then generates burst traffic to synchronize model parameters. We denote rapidly-generated packets for model synchronization as “burst packets,” the time taken to communicate all the burst packets in a single direction (e.g., push and pull) as the “burst interval,” and the time taken from the end of pull to the beginning of the next push as the “network-idle period.” Similarly, the period when GPUs do not train a model is called the "GPU-idle period." Although the size of the burst packet and the length of the network-idle period differ in the deep learning models (i.e., ResNet in Figure 2a, AlexNet in Figure 2b, and VGG16 in Figure 2c), the traffic of all three models shows a similar burst-idle traffic pattern. Also, the results show that the patterns are repeated until the end of the training.

Prior studies [14,15] (not in DDL) have shown that this kind of bursty traffic rapidly fills up switch queues and causes network congestion. Because DDL traffic is bursty, it is also expected to suffer from similar congestion issues, which would result in prolonged training time.

DDL traffic becomes even more bursty due to the following factors: First, the parameters to be synchronized at each iteration are packaged into packets. Most NICs support offloading that transmits packets much faster than the original packet generation rate with the CPU in packet processing. Therefore, from the perspective of network switches, the burstiness becomes severe due to an accelerated NIC offloading [30]. Second, when multiple workers synchronize parameter in PS architecture, the PS becomes an incast point where network traffic is concentrated. This traffic concentration worsens network congestion.

### 2.2. Related Work

In this Section, we review the related work to this study in four categories: (1) host-side scheduling on DDL traffic (Section 2.2.1), (2) reducing the amount of DDL traffic (Section 2.2.2), (3) improvement using an in-network switch (Section 2.2.3), and (4) ECN-based approach (Section 2.2.4). The first three categories are for improving DDL communication while the last, the ECN approach, is for improving network congestion in datacenters, not for DDL traffic.

#### 2.2.1. Host-Side Scheduling on DDL Traffic

**Figure 3:**
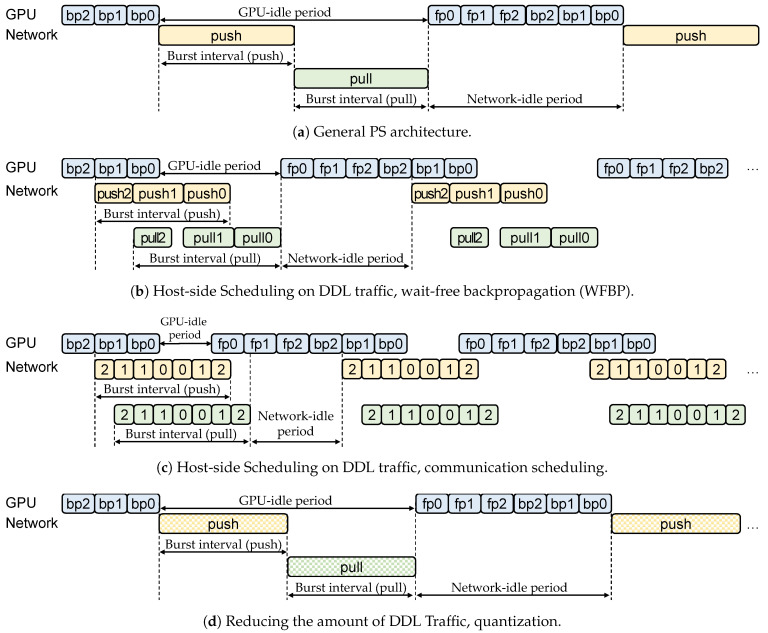
Comparison of communication method of a worker in related work.

DDL usually does not achieve a linear speed-up relative to the number of workers due to the communication overhead [6,7,8]. Specifically, in traditional DDL, each worker performs forward propagation (fp), backpropagation (bp), and push and pull operations sequentially (Figure 3a). These communication operations occupy a large portion of the total training time (communication overhead). Many studies [6,7,8,16,17,18] have attempted to reduce the communication overhead of DDL. Most approaches are based on wait-free backpropagation (WFBP) [8,18] that pipelines the communication and computation of DDL workloads (Figure 3b). Rather than waiting for the end of each training iteration, the communication operation (push and pull) of the *i*-th layer is executed at the end of the backpropagation of the *i*-th layer ($bp_{i}$). So, communication and computation can be performed concurrently. In terms of burstiness, this method reduces the network-idle period (Figure 3b) more than the general PS architecture (Figure 3a), but still, the time required for fp and the first bp (bp2 in Figure 3b) becomes the network-idle period. Also, the number of the burst packets is similar because these methods only divide them when the packets are pushed or pulled by layer. So, the burst characteristic remains.

The communication scheduling approach [6,16,17] further optimizes the WFBP algorithm by scheduling the sending order of each layer in a deep learning model (Figure 3c). WFBP algorithms synchronize parameters from the *n*-th to the 0-th layer because backpropagation calculates layers in reverse order. Therefore, the next iteration waits until the end of the last push and pull operation because the next iteration starts from the 0-th layer. The communication scheduling approach is to schedule the synchronization order according to the operation dependency so that layers to be used sooner are sent earlier. In Figure 3c, we can see that the communication scheduling does not reduce the number of burst packets because it only changes the order of packets, which implies that in host NIC’s or network switches, which are the focus of this paper, the burstiness characteristics remain.

Moreover, although the host-side scheduling approaches divide the burst packets per layer, the divided layer-wise packets are transmitted between the PS and workers in the batched manner to reduce the networking overheads of the divided packets. For example, Tensor Fusion in Horovod [31] sets 64 MB as its default for batching, and ByteScheduler [6] performs run-time batch-size tuning. This packet batching means that at least 64 MB of packets are sent at once, which is sufficient for the PCN to perceive them as burst packets. Furthermore, suppose GPU computation is faster than communication. In that case, the packets will be sent consecutively (e.g., 128 MB and 256 MB), which may cause in-network congestion. The host-side scheduling has nothing to do with it.

#### 2.2.2. Reducing the Amount of DDL Traffic

Another approach that previous studies have taken is reducing the amount of DDL traffic, and the representative methods are quantization [32,33,34] and selective synchronization [35]. First, quantization is a technique to lower the memory usage of model parameters by reducing the precision of the floating-point representation of parameters (e.g., 32 bit to 8 bit) (Figure 3d). They reduce the amount of gradients to be communicated between workers and PS, thereby reducing communication costs. Model quantization is a popular approach for artificial intelligence in mobile and Internet-of-things devices (e.g., TensorFlow Lite [36]).

In addition, Gaia [35] proposed the selective synchronization of gradients, which omits the push and pull operations of the gradients with trivial values that do not change or improve the model by much. These two methods successfully decrease the amount of DDL traffic, but the reduced amount of communication inevitably sacrifices model accuracy and convergence time. In terms of DDL burst traffic (Figure 3d), the number of burst packets could be reduced through quantization or selective synchronization, but they cannot be totally removed. Also, the network-idle period remains similar to the general PS architecture in Figure 3a.

#### 2.2.3. Improvement Using an In-Network Switch

Several studies tried to improve DDL training using in-network switches. First, Geryon [22] proposed network-level flow scheduling for DDL. Host-side scheduling approach (Section 2.2.1) has a limitation that its scheduling scope is on each worker; however, many workers are competing in the network. For example, when packets from other workers contend the bandwidth resource, host-side scheduling has nothing to do. To overcome such limitations, Geryon designs in-network flow scheduling, which makes the parameter of each layer to be synchronized by different flows and gives a two-level priority (i.e., urgent and not urgent) to each flow. Then, the in-network switch processes the packets that belong to each flow according to the given priority so that synchronization of more urgent layers can be done earlier. Geryon can work with host-side scheduling, such as WFBP, but two-level priorities may not be enough because the number of model layers increases rapidly, from tens (e.g., VGG16) to hundreds (e.g., ResNet) and more. Also, the fairness issue always comes with priority.

**Figure 4:**
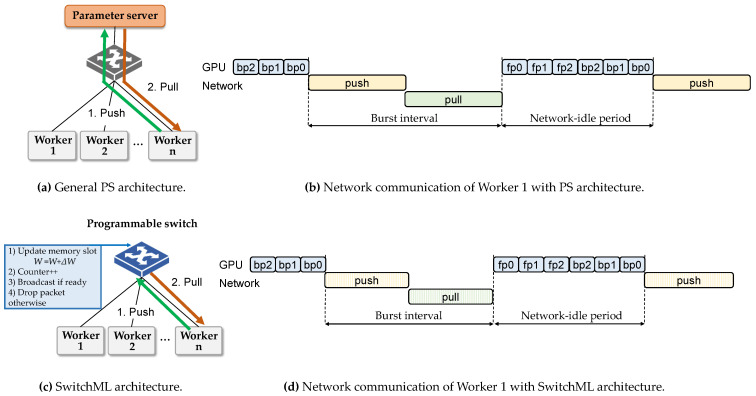
Comparison between PS and SwitchML architectures.

Another recent study, SwitchML [23], used a programmable switch to aggregate the gradients from multiple servers. Compared with the general PS architecture (Figure 4a), SwitchML (Figure 4c) makes the programmable switch perform the role of the PS. Specifically, when each worker sends its gradients, the switch updates its memory slot with the gradients (① in Figure 4c). The switch counts the number of gradients collected (②) to check that the gradients from all workers have been received. Then, the updated model parameters are distributed to all workers by broadcast (③). This process is similar to the pull operation of the general PS architecture. Because the switch takes the role of PS, a packet for push is dropped after the update (④). In this way, DDL traffic for the push and pull operations of each worker goes through fewer network hops than the general PS architecture (e.g., switch to the PS in Figure 4a). Although its limitation is the memory capacity of the current programmable switch, which is of tens of megabytes, much smaller than DDL parameters, it is a promising study that uses a programmable switch for DDL training improvement.

In terms of the DDL traffic pattern (Figure 4b,d), the SwitchML architecture still shows burstiness. SwitchML improves the time to finish push and pull operations using an in-network switch for parameter aggregations. However, the amount of gradients to synchronize does not change. Therefore, the network switch still suffers a packet-burst problem.

One thing to note is that SwitchML mechanism could worsen the DDL traffic burstiness. A SwitchML should parse the packet to extract gradients to be aggregated from each push packet, but today’s programmable switches parse only up to a certain number of bytes in each packet (e.g., 128 MB). Considering an Ethernet frame whose maximum transmission unit (MTU) is 1518 bytes, including headers and each gradient element (the model gradient is expressed in a matrix form and each number that constitutes the matrix is a gradient element.), are 32-bit floating point value, the MTU-size packet can include up to 366 elements as discussed in SwitchML paper [23]; however, packets in SwitchML contain only 32 elements per packet. This means that to transmit the same number of gradients, SwitchML requires a number of packets 11.4 times higher than the general PS architecture, thus causing the switch’s queue to be filled quickly and worsening the burstiness. As the authors of the SwitchML pointed out, the packet loss handling and congestion control for DDL traffic should be solved. We believe PCN could potentially relieve packet losses and network congestion using a programmable switch.

#### 2.2.4. ECN-Based Approach

Apart from studies to improve DDL communication, existing studies handle burst traffic for datacenters [14,15]. They mostly use explicit congestion notification (ECN). When the switch detects that its queue is nearly full, burst traffic is dropped or suffers a large delay. The detection of such congestion is done by a fixed threshold value of the switch queue. If the enqueued packets exceed the threshold, the switch sets a congestion bit (congestion mark) in the IP header. The receiver of the ECN marked packet acknowledges the sender by marking an ECN-echo flag in the TCP header so that the sender takes note of the existence of congestion and reduces its sending rate.

Datacenter TCP (DCTCP) [14] is an improved transport layer protocol that notifies the extent of congestion using ECN. DCTCP senders maintain estimates of the packets marked and adjust their congestion windows accordingly. While ECN marks congestion based on queue length at packet enqueue, ECN* [15] improves burst tolerance without modifying the TCP congestion control by simply marking congestion using queue length at packet dequeue. Because congestion changes while packets are buffered in a queue, congestion marking at dequeue can deliver a more accurate congestion state. These two approaches improve latency and throughput.

While ECN-based approaches for datacenters can effectively mitigate congestion and reduce latency, DDL workloads and their burstiness are not within its scope. Because the general traffic in datacenters is unpredictable in its burstiness, these approaches work reactively. This means that handling the congestion is done after the congestion takes place, so the network is not ready for the burst traffic. In contrast, since DDL traffic shows periodical burstiness, we propose PCN that prepares for burst traffic proactively.

#### 2.2.5. Novelty of PCN

The novelties of PCN with regard to existing studies are as follows. Host-side scheduling approach reduces the communication overhead of DDL traffic, but congestion issues remain. Reducing the amount of DDL traffic improves congestion and burstiness issues in some degrees because the total amount of generated traffic is reduced but sacrificing model accuracy may not be acceptable to many researchers. For in-network flow scheduling, Geryon has only two priority levels, so fine-grained scheduling is not possible yet. SwitchML achieves in-network aggregation, yet it recognizes that lack of in-network congestion control can slow down overall performance. Thus, PCN, which attempts to prevent congestion in-network, can help the congestion in existing studies. To the best of our knowledge, PCN is the first work that proactively solves network congestion problems caused by the burstiness of DDL traffic.

### 2.3. P4 and Switch Programmability

**Figure 5:**
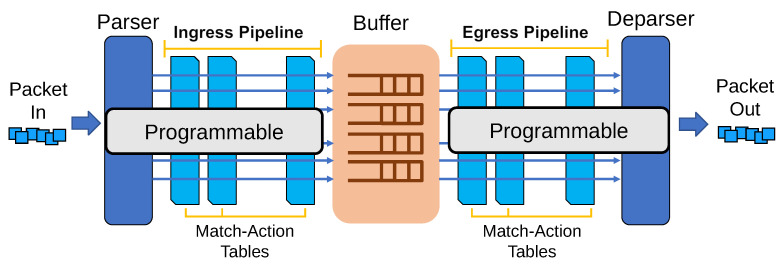
P4 architecture.

P4 [21] is a high-level language designed to program packet processors, especially for programmable switches. Figure 5 shows protocol independent switch architecture (PISA) [37] and its programmable parts. First, a switch parses the packet header (e.g., TCP, IP, and Ethernet) when a new packet is received (Packet In). After using the parsed header fields and match-action tables, the switch determines the action to apply to the packet (e.g., set an outgoing port). Buffers exist for each outgoing port (multiple buffers may exist on each port for priority queueing), and packets are queued in a buffer based on an outgoing port determined by an ingress pipeline. Then, packets are processed in an egress pipeline, concatenated with parsed headers to the packet at the deparser and, finally, sent out to an outgoing port. A key aspect of P4 is that it enables network operators to change how a switch processes packets (“programmable” in Figure 5).

The goal of PCN is to regulate the switch’s queue length to handle the burst packets. To do that, PCN requires switches to parse a new type of packet header, update their thresholds according to the new packet header fields and apply thresholds to queues that share the same outgoing port with burst packets. This is not possible with traditional switches, including SDN switches [21], because they only support a fixed set of functions that cannot be modified. Hence, we use P4 to implement custom packet processing logics on programmable switches. When using P4, we define the PCN-START packet that delivers the PCN threshold in header fields and implement a switch operation that changes the initial threshold to the PCN threshold when the switch receives the PCN-START packet.

## 3. Design

PCN is a congestion avoidance technique that utilizes P4 programmable switches. PCN lowers queue length within a switch before burst traffic arrives (details in Section 3.2). To make a switch prepare for the burst packets, PCN notifies the network switches that the burst packets will be generated. We explain PCN operations as follows. First, the communication sequence for PCN is explained (Section 3.1). Then, the detailed operations of the network switch are described (Section 3.2). In addition, the policy for deciding the PCN threshold is explained (Section 3.3).

### 3.1. Communication Sequence between Worker and PS

**Figure 6:**
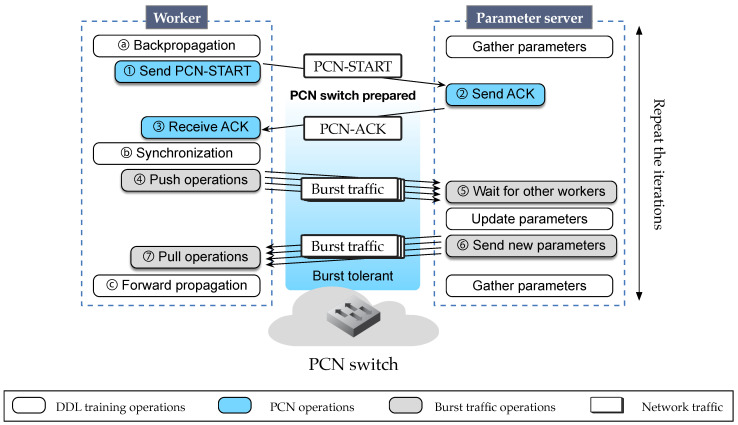
Communication between worker and PS.

In PS architecture, each worker performs backpropagation (ⓐ in Figure 6) on the given mini-batch inputs. At the end of each backpropagation, the worker sends and receives burst traffic with PS (ⓑ). In PCN, the workers send the PCN-START packet to the PS before sending burst traffic (① in Figure 6). The PCN-START packet is sent to PS, not to a specific switch, because multiple switches may exist in the path between the worker and PS. By sending one PCN-START packet to PS, the PCN switches between the worker and PS apply the PCN threshold; hence, we can avoid sending multiple PCN-START packets individually to each PCN switch. In addition, workers do not know the network addresses of PCN switches directly, so it is efficient to make the workers send PCN-START to PS rather than to PCN switches.

Then, the PCN-START packet traverses all the network switches between the worker and the PS, so the switches are notified of the upcoming burst traffic. The PCN-START packet contains a PCN threshold, a new queue threshold of a switch for the burst traffic. The PCN threshold is explicitly determined by the DDL operator (executor of workers and PS) so the PCN thresholds sent by the workers are identical. We explain how to decide the threshold value in Section 3.3.

When receiving the PCN-START, the PS sends ACK for the PCN-START packet (②) so that the worker starts to generate the burst packets containing model gradients of the deep learning models (④). PCN switches have a time window of one round-trip-time (RTT) for the PCN-START packet and its ACK packet to get prepared for the burst traffic. When gradients from every worker are received (⑤), the PS updates the model parameters and broadcasts the updated values to all workers (⑥, ⑦).

**Figure 7:**
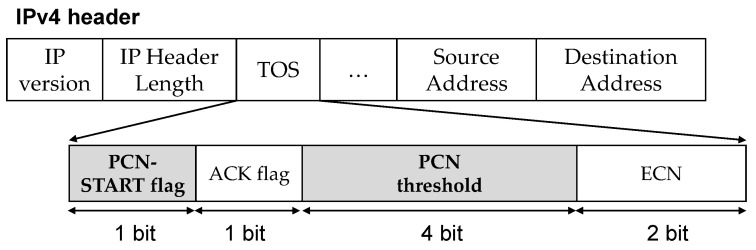
PCN-START packet header.

We design the PCN-START packet to use Type-of-Service (TOS) fields in the IPv4 header to carry information needed for PCN (Figure 7). TOS field consists of 6 bits of DSCP fields and 2 bits of ECN fields. We use the first 2 bits of DSCP field as the PCN-START flag (1 bit) and ACK flag (1 bit), and the other 4 bits for expressing the PCN threshold. Since most switches’ thresholds are usually multiple of 5 and lower than 80 [14,15], we enter a PCN threshold divided by five into the remaining four bits. If DSCP is used by other protocols, we can use IP options fields instead.

### 3.2. Switch Operation for PCN

**Figure 8:**
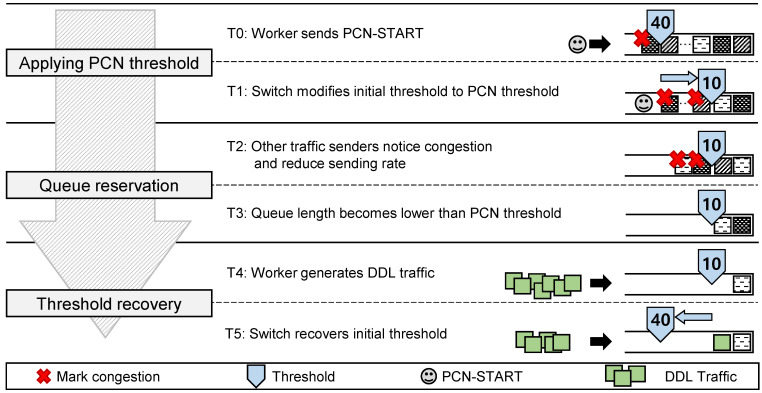
Switch operation for PCN.

In this section, we explain the switch operations for PCN (Figure 8) in three steps: (1) applying the PCN threshold, (2) queue reservation, and (3) threshold recovery. We also discuss how the switch queue state changes in each step.

- Applying the PCN threshold: When a worker sends the PCN-START packet (T0 in Figure 8), the switch parses the PCN-START header and obtains the PCN threshold. Then, the switch saves the PCN threshold in the switch register and applies the PCN threshold for its queue (T1), instead of the current queue threshold (initial threshold). The PCN threshold should be smaller than the initial threshold in order to prepare for the burst DDL traffic (details in Section 3.3). In case when multiple workers send the PCN-START packets at the same time, the switch manages the counter, increases the counter every time the PCN-START packet arrives, and decreases the counter every time the burst DDL traffic arrives. The PCN threshold is applied when the counter is non-zero.
- Queue reservation: Queue length tends to fluctuate around the threshold value. For example, if the initial threshold is 40, the queue length usually fluctuates from 25 to 50 in our environment, showing a saw-tooth pattern because of host-side congestion control. In this situation, when the PCN threshold is applied (for example, 10), packets enqueued at the switch (from 10th to the last packets) are considered as the ones that cause network congestion. So, since the initial threshold is larger than the PCN threshold, a number of packets (higher than PCN threshold) occupy the switch’s queue when the PCN threshold is applied. Incoming packets are then marked as congested, and the packets’ senders get notified of congestion. So, the senders slow down their packet sending rates according to the TCP/IP protocol (T2). Thus, the switch’s queue length becomes lower than the PCN threshold (T3), which makes the switch more burst tolerable.
- Threshold recovery: The time between T0 and T3 takes about one RTT because the queue length gets changed to the PCN threshold when senders change their sending rates. So, in T4, workers need to start the burst DDL traffic after one RTT from sending the PCN-START packet so that the switch is ready for the burstiness. This scheme is achieved by workers that generate DDL traffic after receiving an ACK packet for the PCN-START packet from the PS. Then, the DDL traffic is processed in the switch. Once the DDL traffic arrives at the switch, in T5, PCN restores the threshold back to the initial threshold in order to recover the sending rates. While DDL traffic is being processed, the background traffic also recovers its sending rate. This may cause another congestion to the DDL traffic. However, because the DDL traffic is already being queued earlier than the background traffic, and the background traffic senders increase sending rates slowly (according to TCP additive increase), the chance for this congestion is low. If it happens, the switch can use the ECN approach to slow down the sender of background traffic. The rationale of T5 is to minimize the throughput reduction, and the throughput reduction will be measured in Section 4.4.

### 3.3. PCN Threshold Policy

A high threshold leads to high throughput for long flow, whereas a lower threshold leads to low latency and high burst tolerance for DDL traffic. PCN temporarily reduces the threshold to achieve burst tolerance and immediately recovers the threshold after burst traffic arrival to achieve high throughput. However, setting the threshold too low can lead to poor network throughput. A prior study [4] formulated the lower bound of the ECN threshold without throughput degradation, as defined in Equation (1). It is widely known that *N* (the number of concurrent long flows) in Equation (1) is challenging to determine exactly. Thus, prior studies [14,15] instead use an average number (which is approximately three) of long TCP connections on each bottleneck link from datacenter packet traces. Thus, we also set *N* to three when setting the ECN threshold. Also, using metrics, such as RTT, MTU, and link capacity (C), measured in an evaluation environment (Section 4), we find $\frac{RTT\times C}{\sqrt{N}\times MTU}=37$. Therefore, we set our initial threshold ($h_{initial}$) as 40 (ECN threshold is usually a multiple of 5).

(1) $$h_{initial}>\frac{RTT\times C}{\sqrt{N}\times MTU},$$

Following this, we set the PCN threshold ($h_{PCN}$) using $h_{initial}$ and the appropriate ratio (*k*), as in Equation (2). This ratio determines the amount of switch queue reservation. For example, if we set *k* as 1/2 given $\frac{RTT\times C}{\sqrt{N}\times MTU}=37$, we obtain $h_{PCN}$ of 20, and the switch queue length reduces until it reaches 20. If *k* is set to 1, then applying PCN will not change anything, as $h_{initial}$ = $h_{PCN}$. When the value of *k* decreases, more space in the queue is reserved for the burst DDL packets; in short, the smaller ratio leads to a smaller PCN threshold and high burst tolerance. We empirically choose 1/4 for *k* in the evaluation, considering the degree of burst tolerance needed; thus, $h_{PCN}$ is set to 10, and the reserved switch queue size becomes (queue capacity − 10).

(2) $$h_{PCN}=\frac{RTT\times C}{\sqrt{N}\times MTU}\times k,$$

## 4. Evaluation

In this section, we show the evaluation results of our proof-of-concept implementation. The PCN mechanism is implemented in two parts: (1) DDL traffic generator that sends PCN-START and simulates DDL traffic, and (2) P4 switch that implements PCN.

We implement PCN in P4 and evaluate using BMv2 [24], a P4 software switch. We set a tree network topology of a single root and two leaf switches, a frequently used topology. As discussed in Section 2.1 and Figure 2, DDL traffic differs in its amount and interval for each model, but the burst-idle traffic pattern is common among all the models. Therefore, instead of using individual models, we implement a DDL traffic generator called DDLgen that simulates this burst-idle traffic pattern generated by a worker, based on tcpreplay [38] and iperf3 [39]. As a baseline, DDLgen generates burst traffic using iperf3 burst mode and stays idle for the given network-idle period. DDLgen repeats this pattern similar to the burst-interval traffic pattern observed (Section 2.1). To evaluate the DDL traffic performance with PCN design, we add PCN operations to DDLgen, which means that DDLgen first sends the PCN-START packet, waits for the ACK packet, and repeats the burst-interval pattern as explained in Figure 6.

We run DDLgen with and without PCN, measure performance improvement, and monitor the switch queue status. For each leaf switch, three hosts generate background traffic (in datacenters, the number of concurrent long TCP connections to a server is generally two to three [3].) for 100 s using iperf3. We run DDLgen to generate DDL traffic after 20 s of background traffic in order to make the background traffic stable (because of TCP’s congestion control (e.g., slow start, congestion window), TCP throughput fluctuates at the beginning and becomes stable later. The background traffic becomes stabilized after 20 s in our environment). DDLgen simulates six iterations of burst-idle traffic patterns with a 7 s network-idle period. We use initial and PCN thresholds of 40 and 10, respectively, following the policy stated in Section 3.3. Our switch in evaluations has a queue with a length of 60, so the queue length ranges from 0 to 59.

PCN is the first approach to improve the network IO bottleneck by using programmable switches. Whereas existing studies (Section 2.2) deal with optimizations on hosts (i.e., workers and PS), PCN is implemented in network switches; thus, the techniques are complementary to each other. In other words, it is difficult to compare the existing studies with PCN (for queueing latency or queue length metrics). Thus, we evaluate PCN when it is turned on or off.

### 4.1. Evaluation Metrics

We conduct evaluations on the three key categories: (1) queue length change (Section 4.2), (2) performance improvement (Section 4.3), and (3) overheads (Section 4.4). The detailed measurement of the three categories are as follows:

- Queue length change: We measure the queue length of a switch as the preparedness for the burst packets of PCN. Two measurements, start queue length and maximum queue length, are measured. Start queue length represents the queue length occupied by the background packets before the DDL packets arrive at the switch, which shows the switch queue status right before the burst packets of the iteration arrive. The maximum queue length is the highest switch queue length during each burst interval.
- Performance improvement: To evaluate the performance improvement by PCN, we use three metrics–average throughput, burst completion time, and queueing latency. The average throughput is the amount of transmitted network traffic, and the burst completion time refers to the time taken for network communication of burst packets to be completed. Also, queueing latency is per-packet latency in a network switch caused by the existing packets in the switch queue. These three metrics are measured and compared with and without running PCN. To measure the queueing latency, we implement in-band telemetry [40] that makes the packets contain custom network statistics, such as queueing latency per switch.
- Overheads: PCN makes the switch available for burst packets by reducing its queue threshold. Although this scheme is effective on DDL packets, it could reduce the throughput of other background traffic. So, we measure the decreased throughput of background traffic as an overhead of PCN.

### 4.2. Queue Length Change

**Figure 9:**
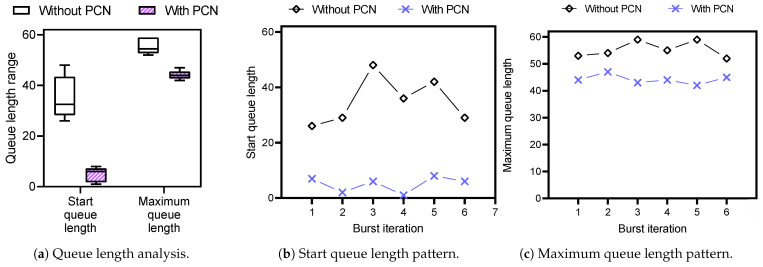
Burst tolerance.

To see the improved burst tolerance with PCN, we evaluate the queue length change by measuring (1) start queue length and (2) maximum queue length. We repeat the experiment more than 20 times to gain reliable and reasonable results. Figure 9a shows the distribution of the queue lengths as box plots which present the median values at their middle and the minimum and maximum values using the bars. Also, Figure 9b and c each show the specific pattern of start queue length and maximum queue length, respectively, during six burst iterations generated by DDLgen.

First, without PCN, the start queue length when the first packet of a burst is enqueued ranges from 26 to 48. On the other hand, PCN ranges from 1 to 8. On average, the start queue length is 35 without PCN and 5 with PCN. With a queue capacity of 60, a switch queue without PCN has space for 25 incoming packets, while a switch queue with PCN has space for 55 incoming packets. Therefore, PCN enables the switch to handle twice as many burst packets.

In terms of the maximum queue length, the queue lengths without PCN range from 52 to 59, while the ones with PCN range from 42 to 47. On average, without PCN case shows 54.8 of queue length, and frequently reaches up to 59, which means that the switch queue becomes full of packets. This indicates that incoming burst packets will be dropped without PCN. With PCN, the maximum queue length is 44.1 on average, and queue length does not go over 50, indicating that it neither causes packet loss nor congestion.

### 4.3. Performance Improvement

**Figure 10:**
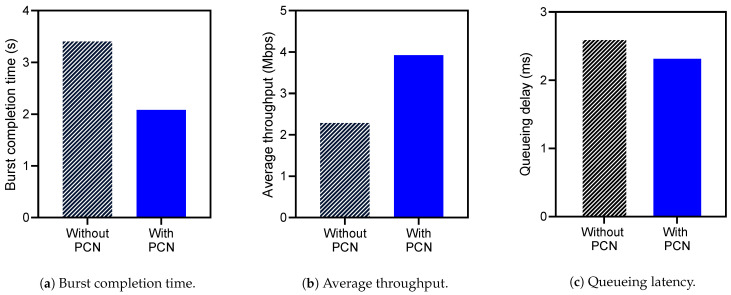
Performance improvement.

Firstly, Figure 10a,b show the average burst completion time and the throughput of DDL traffic, respectively, when the PCN is on or off. The results indicate that PCN reduces the burst completion time by 39% (Figure 10a) and improves throughput by 72% (Figure 10b). Such improvements result from PCN that reserves a portion of a packet queue for burst packets of DDL workload in advance; so with PCN, DDL traffic’s sending rate increases properly with a spacious switch queue. On the contrary, without PCN, DDL traffic suffers network congestion due to the deficient queue space. In addition, we measure the per-packet queueing latency within a switch (Figure 10c). With PCN, the average queueing latency of packets processed during burst intervals is reduced by 13%. These improvements show that PCN appropriately mitigates the congestion caused by DDL traffic.

### 4.4. Overheads

Here, we investigate the overhead of the PCN design. We see the throughput of the background traffic because PCN reduces all traffic sending rates before the DDL burst impacts the background traffic throughput. In our results, the background traffic throughput decreases by approximately 5.7%. PCN does not preempt the background traffic but reduces the sending rate of the switch while the PCN threshold is applied. Note that this overhead is transient and disappears by the PCN threshold recovery. As the overhead of PCN, PCN-START is added in ordinary PS architecture for delivering the PCN threshold to the switches. The amount of traffic increased by PCN-START is a single packet without any payload (approximately 64 bytes), which means the overhead is negligible. In addition, to identify the PCN-START packet and deliver the PCN threshold, 6 bits of DSCP fields in the IP header are used.

## 5. Discussion

### 5.1. Estimation of the Total DDL Training Time

In this paper, we show that PCN improves throughput, queueing latency, and queue length changes in DDL traffic. Now the following question remains: how the improvement helps the total DDL training time. Here we estimate the total training time of the DDL workload under the general PS architecture. Because network communication is only a part of DDL training, we estimate by factoring the degree of improvement by PCN in the total DDL training time. We formulate our estimation with the ratio of throughput without PCN ($t_{w/oPCN}$) to throughput with PCN ($t_{wPCN}$), as expressed by Equation (3) where ρ is the portion of communication time.

(3) $$\rho \times \left(1-\frac{t_{w/oPCN}}{t_{wPCN}}\right),$$

According to prior studies [7], ResNet32, AlexNet, VGG16, and Inception-v3 have ρ of 47.8%, 85.2%, 92.8%, and 64.5%, respectively. Applying these values to Equation (3), the estimated improvements in the total DDL training time of ResNet32, AlexNet, VGG16, and Inception-v3 are 20%, 35.6%, 38.8%, and 27.0%, respectively.

### 5.2. The Impact of Environment Changes on PCN

In this paper, we evaluate (Section 4) and estimate (Section 5.1) the performance of PCN under the value ρ from another paper [7]. One may wonder whether the results depend on the computing environment, such as mini-batch size, GPU version, network bandwidth, and network topology. Although most of the evaluation setting factors could change indicators like ρ, we believe that the effectiveness of PCN can still stand due to the following reasons. First, in terms of the mini-batch size, the large mini-batch size prolongs the computing time (network-idle period). However, the total number of parameters to synchronize does not change. Thus, the burst characteristics of the traffic remain. Second, similar to the mini-batch, the GPU version affects the training time, not the traffic characteristics.

Third, network bandwidth determines network congestion. Suppose that the network links between the workers and PS support up to 100 Gbps, which is common in datacenters nowadays. Considering that the worker consumes 10 Gbps of network traffic for DDL communications (shown in the evaluation of Figure 6), and up to 256 workers (with 2048 GPUs in total) can operate in a large-scale DDL environment [41], the switches between the workers and PS suffer from network congestion. Thus, PCN’s approach, which avoids congestion caused by DDL burst traffic, is still practical when the network bandwidth becomes higher than the one used in our evaluations. In addition, PCN works regardless of the type of network topology because PCN solves the congestion caused in a specific port within a switch. In other words, irrespective of the topology, PCN is valid in switch queues with congestion caused by traffic burstiness.

### 5.3. Multi-Tenancy

**Figure 11:**
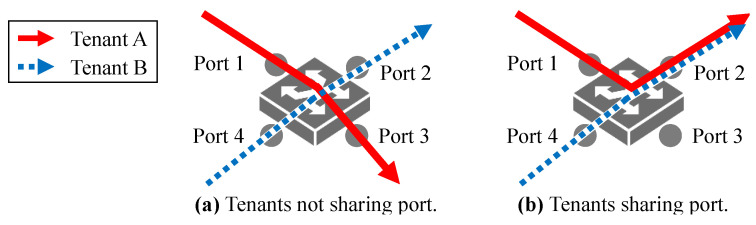
Multi-tenant scenarios.

When multi-tenants exist, where many users run their own DDL workloads on a shared infrastructure (e.g., cloud computing [42,43,44,45]), an issue that PCN has to solve is how to handle the multiple PCN thresholds of different tenants because a switch port’s queue can operate only under a single threshold [19]. This issue can be categorized into two scenarios: (1) the PCN switch’s port is not shared (Figure 11a), and (2) the PCN switch’s port is shared (Figure 11b). For the not-shared scenario, each switch port is associated with one tenant, so the switch port is applied by a PCN threshold. So, this scenario is not an issue for PCN. On the other hand, if tenants share a port at the same time (Port 2 in Figure 11b), PCN needs another mechanism because a port can only work with a single PCN threshold. We leave this as future research.

## 6. Conclusions

This paper presents a new technique called PCN that prevents network congestion caused by DDL burst traffic. PCN proactively makes room in the switch queue so that the network can handle the burst DDL traffic. In our evaluation, PCN improves the burst throughput by 72%, and the switch can handle two times more DDL packets on average without causing network congestion. As the future directions, we believe that the integration with other DDL optimizations (e.g., Horovod and ByteScheduler) would be essential. Second, considerations about multi-tenancy, which has been explained in Section 5.3, should be addressed. Third, wide-scale evaluations on DDL infrastructures (e.g., GPU cluster) would be helpful for the validation.

## Data Availability

The data presented in this study are available on request from the corresponding author.
